# On two new species of the genus *Afissa* Dieke, 1947 (Coccinellidae, Epilachnini) from southwestern China

**DOI:** 10.3897/zookeys.1291.193001

**Published:** 2026-09-01

**Authors:** Xinning Zhang, Zihao Zhu, Xingmin Wang

**Affiliations:** 1 South Campus, Shenzhen Senior High School, Shenzhen 518000, China South Campus, Shenzhen Senior High School Shenzhen China; 2 Department of Entomology, College of Plant Protection, South China Agricultural University, Guangzhou 510642, China Department of Entomology, College of Plant Protection, South China Agricultural University Guangzhou China; 3 Engineering Research Center of Biological Control, Ministry of Education, Guangzhou 510640, China Engineering Research Center of Biological Control, Ministry of Education Guangzhou China

**Keywords:** *
Afissa
*, Coccinellinae, distribution, ladybird beetle, new species, redescription, taxonomy

## Abstract

Two new species of the genus *Afissa* Dieke, 1947 from southwestern China were described and illustrated herein, namely *Afissa parabicrescens* Zhang, Zhu & Wang, **sp. nov**. and *Afissa longa* Zhang, Zhu & Wang, **sp. nov**. *Afissa bicrescens* Dieke, 1947, **stat. rev**. is redescribed, and a short discussion on *Afissa gedeensis* Dieke, 1947 is provided. A distribution map of four *Afissa* species is provided along with illustrations of geographical variation in the tegmen.

## Introduction

The taxonomically difficult, herbivorous ladybird beetle tribe Epilachnini currently includes 27 genera and over 1000 species and has a long and complex nomenclatural history (Tomaszewska and Szawaryn 2016). The latest phylogenetic study of Epilachnini was conducted by Szawaryn et al. (2015), which revealed that several genera are polyphyletic or paraphyletic. Subsequently, Tomaszewska and Szawaryn (2016) revised the world genera of Epilachnini, recognizing nine genera in the Asian fauna. The Chinese fauna of Epilachnini currently comprises approximately 150 species; most of them have been placed in the genus *Epilachna* (Wang and Chen 2022). However, Tomaszewska and Szawaryn (2016) redefined *Epilachna* as being restricted to the New World, indicating that numerous nomenclatural changes will be required in the future.

Dieke (1947) erected the genus *Afissa* with the type species *Coccinella flavicollis* Thunberg, 1781 [= *Afissa flavicollis* (Thunberg, 1781)], characterized by the undivided sixth female ventrite and toothless claws. Considering the similarity between *Afissa flavicollis* and *Epilachna borealis* (Fabricius, 1775) (type species of *Epilachna*), Li and Cook (1961) subsequently synonymized *Afissa* with *Epilachna* Chevrolat in Dejean, 1836. Based on phylogenetic evidence, Szawaryn et al. (2015) resurrected *Afissa* from synonymy with *Epilachna* and proposed that most Asian species of the former *Epilachna* belong to this genus.

To date, 30 species of *Afissa* have been recorded from China (Mulsant 1850; Crotch 1874; Weise 1889; Sicard 1913; Weise 1923; Dieke 1947; Kapur 1955; Li and Cook 1961; Kapur 1963; Bielawski 1967a; Bielawski 1967b; Pang and Mao 1977, 1979; Cao and Xiao 1984; Cao and Wang 1992; Zeng and Yang 1996a; Zeng and Yang 1996b; Yu 1999; Yu 2004; Ren et al. 2009; Pang et al. 2012; Szawaryn et al. 2015; Tomaszewska and Szawaryn 2016; Poorani and Rojeet 2019; Das et al. 2020; Wang and Chen 2022; Das et al. 2023; Iqbal et al. 2025; Wang et al. 2025). However, the taxonomy and diversity of this genus in China remain insufficiently studied, and the identity of many species formerly assigned to *Epilachna* requires further examination. During recent investigations of Chinese Epilachnini, additional specimens referable to *Afissa* were examined. In this paper, two new species of *Afissa* from southwestern China are described and illustrated, and one known species is redescribed.

## Material and methods

All studied materials were preserved in the collection of the Department of Entomology, South China Agricultural University (**SCAU**), Guangzhou, China.

The classification of Epilachnini follows Tomaszewska and Szawaryn (2016). External morphology was examined using a micrometer attached to a SteREO Discovery V20 dissecting stereoscope. The morphological terminology follows Ślipiński (2007). The following measurements were taken: **TL** – total length, from the apical margin of the clypeus to the apex of the elytra; **TW** – total width, across both elytra at the widest part; **TH** – total height, through the highest point of the elytra to the metaventrite; **HW** – head width, including eyes; **PL** – pronotal length, from the middle of the anterior margin to the base of pronotum; **PW** – pronotal width at the widest part; **EL** – elytral length along the suture, from the apex to the base including the scutellar shield.

The abdomen was detached and cleared in warm 10% NaOH solution for several minutes. Genitalia of both sexes were dissected, rinsed with distilled water, transferred to glycerol, and examined on slides. Photographs were taken using digital cameras (ZEISS Imager M2 and a ZEISS Axiocam 506 Color) attached to a dissecting microscope using ZEISS ZEN 2.3 software. Photos were cleaned up and laid out in plates with Adobe Photoshop ver. 26.1.0. Photographs of the habitus were taken using a Nikon Z7 II camera with a Laowa 25 mm f/2.8 2.5–5× Ultra Macro lens. All photographs were processed using Helicon Remote (ver. 3.9.7W) and Helicon Focus ver. 7.0.2 software.

## Taxonomy

### Genus Afissa

Taxon classificationAnimaliaColeopteraCoccinellidae

Dieke, 1947

5CD422D6-3A7C-5FFA-A5C8-83765AA2BBBF

Afissa Dieke, 1947: 113. Type species: Coccinella flavicollis Thunberg, 1781 (by original designation). Type locality: East Indies. Synonymized with Epilachna Chevrolat in Dejean, 1836 by Li and Cook 1961: 51. Treated as a valid genus by Szawaryn et al. 2015: 565.
Afissula Kapur, 1955. Type species: Afissula rana (Kapur, 1955) (by original designation). Type locality: Nepal, Syabrabensi. Synonymized with Afissa Dieke, 1947 by Szawaryn et al. 2015: 565.

#### Diagnostic features.

**Figure 1.**
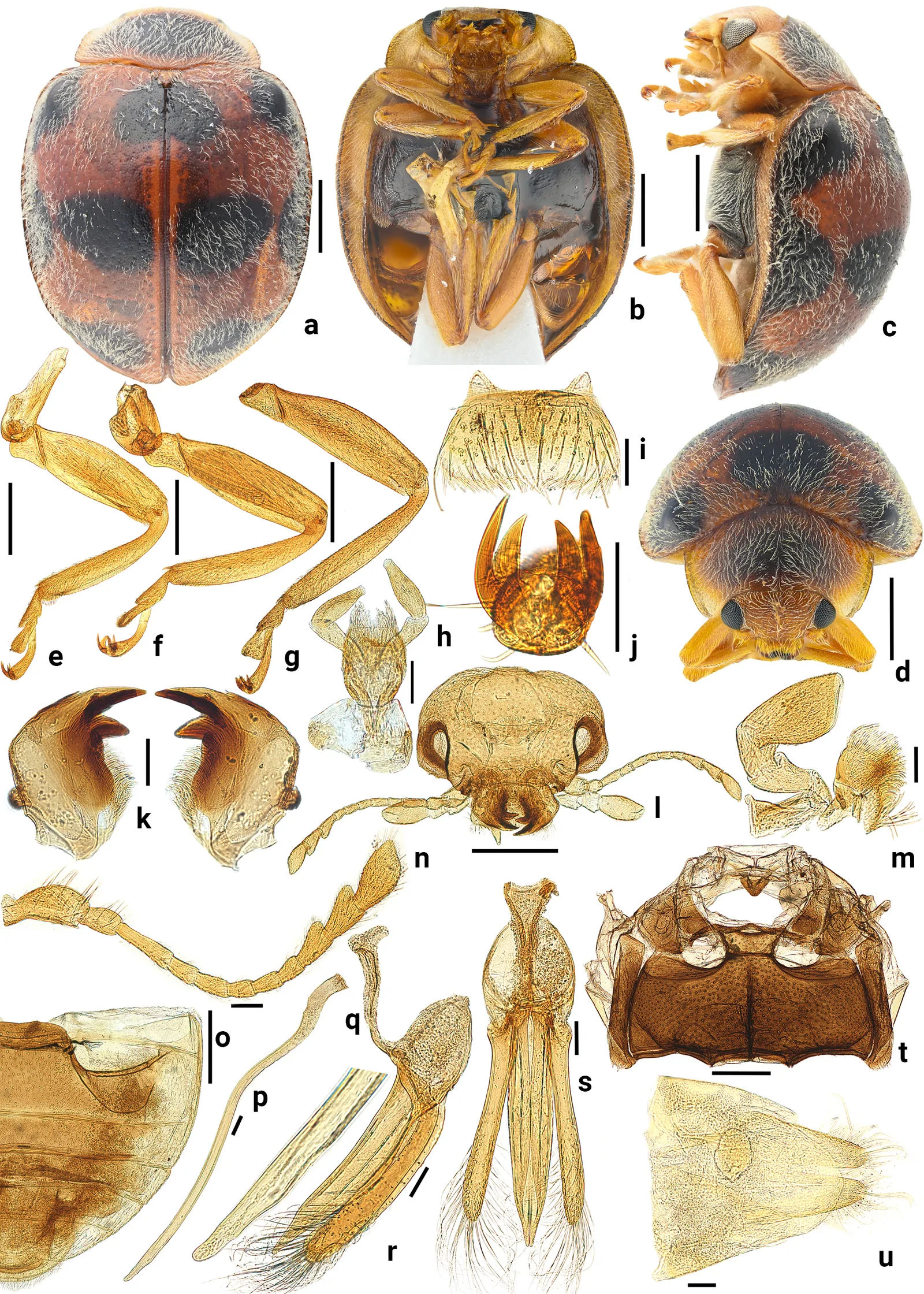
*Afissa parabicrescens* sp. nov. **a–d**. Habitus; **a**. Dorsal view; **b**. Ventral view; **c**. Lateral view; **d**. Frontal view; **e**. Front leg; **f**. Middle leg; **g**. Hind leg; **h**. Labium; **i**. Labrum; **j**. Tarsal claw; **k**. Mandibles; **l**. Head; **m**. Maxilla; **n**. Antenna; **o**. Abdomen; **p**. Penis; **q**. Penis apex; **r**. Tegmen, lateral view; **s**. Tegmen, inner view; **t**. Meso- and metaventrite; **u**. Coxites. Scale bars: 1.0 mm (**a–d**); 0.5 mm (**e–g, l, o, t**); 0.1 mm (**h–k, m, n, p, r, s, u**).

**Figure 2.**
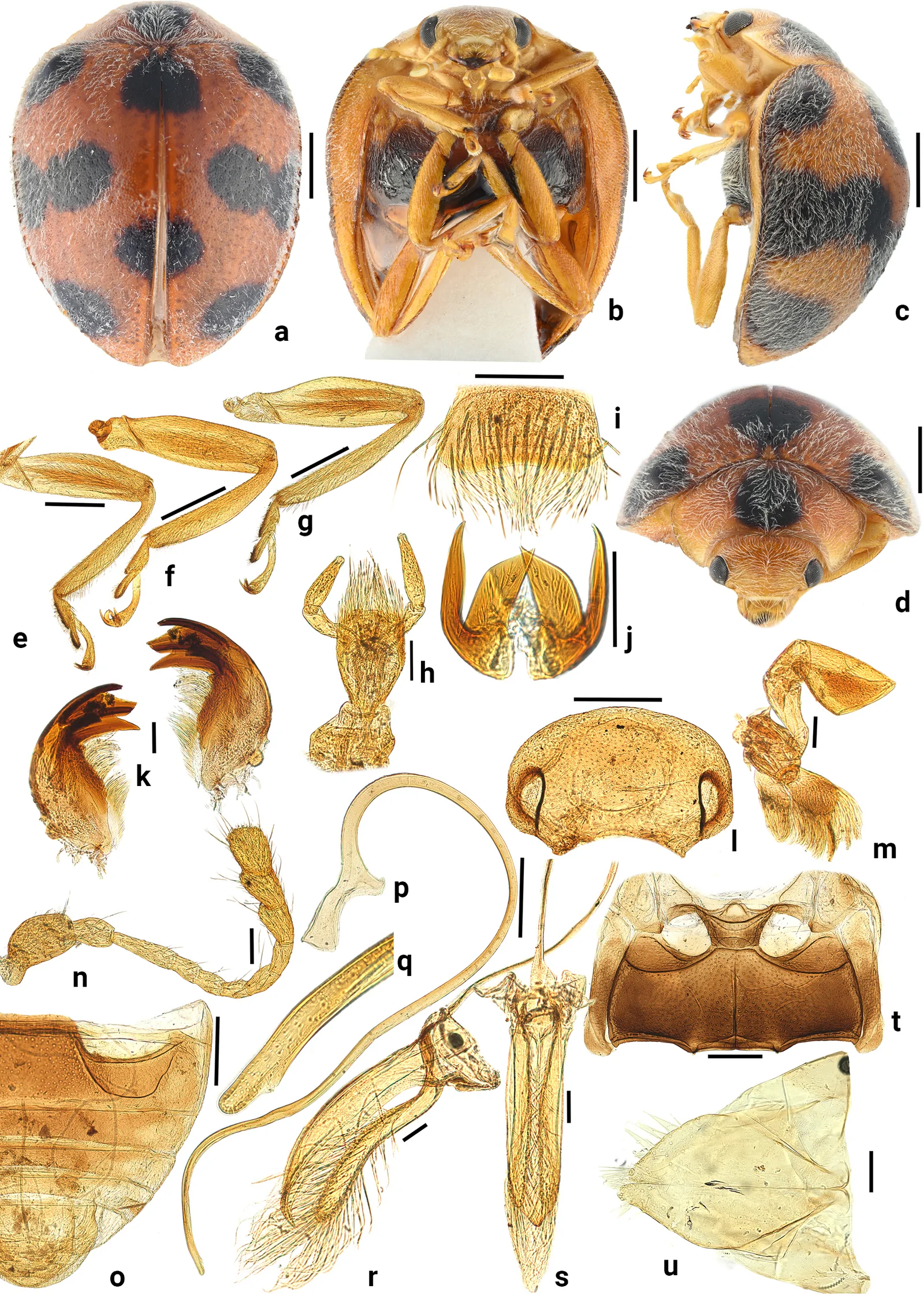
*Afissa longa* sp. nov. **a–d**. Habitus; **a**. Dorsal view; **b**. Ventral view; **c**. Lateral view; **d**. Frontal view; **e**. Front leg; **f**. Middle leg; **g**. Hind leg; **h**. Labium; **i**. Labrum; **j**. Tarsal claw; **k**. Mandibles; **l**. Head; **m**. Maxilla; **n**. Antenna; **o**. Abdomen; **p**. Penis; **q**. Penis apex; **r**. Tegmen, lateral view; **s**. Tegmen, inner view; **t**. Meso- and metaventrite; **u**. Coxites. Scale bars: 1.0 mm (**a–d**); 0.5 mm (**e–g, l, o, t**); 0.1 mm (**h–k, m, n, p, r, s, u**).

**Figure 3.**
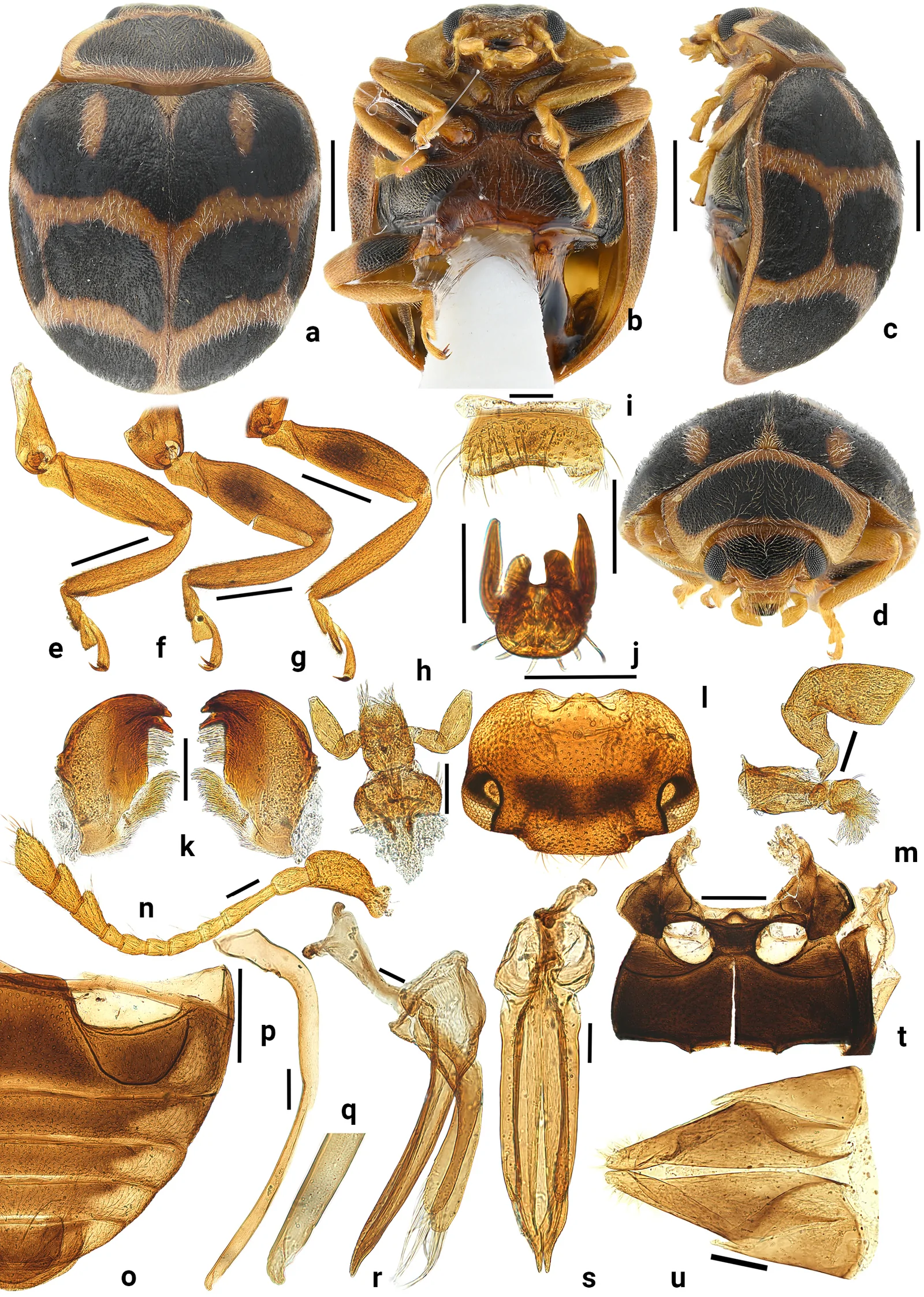
*Afissa bicrescens*. **a–d**. Habitus; **a**. Dorsal view; **b**. Ventral view; **c**. Lateral view; **d**. Frontal view; **e**. Front leg; **f**. Middle leg; **g**. Hind leg; **h**. Labium; **i**. Labrum; **j**. Tarsal claw; **k**. Mandibles; **l**. Head; **m**. Maxilla; **n**. Antenna; **o**. Abdomen; **p**. Penis; **q**. Penis apex; **r**. Tegmen, lateral view; **s**. Tegmen, inner view; **t**. Meso- and metaventrite; **u**. Coxites. Scale bars: 1.0 mm (**a–d**); 0.5 mm (**e–g, l, o, t**); 0.1 mm (**h–k, m, n, p, r, s, u**).

**Figure 4.**
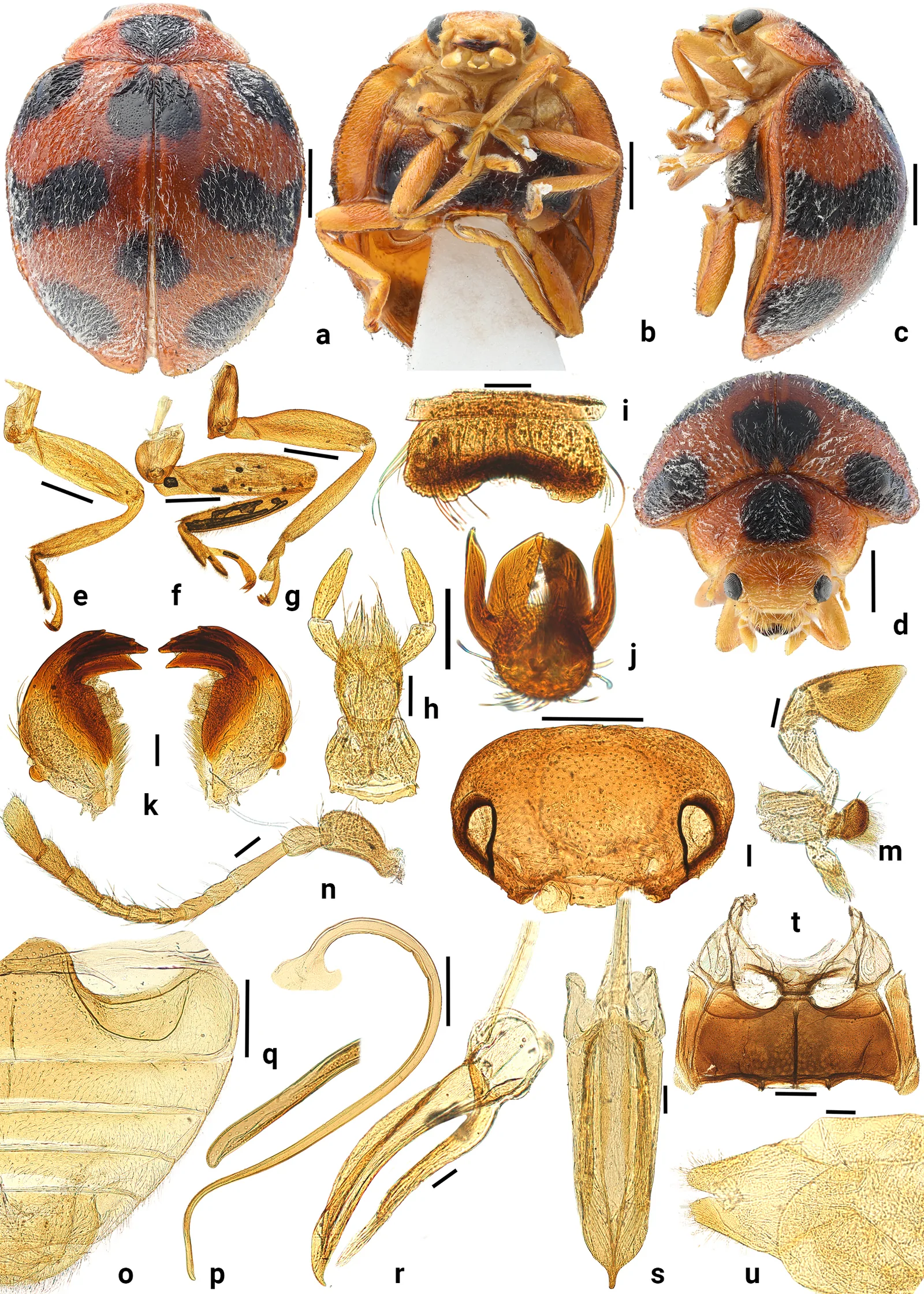
Afissa cf. gedeensis. **a–d**. Habitus; **a**. Dorsal view; **b**. Ventral view; **c**. Lateral view; **d**. Frontal view; **e**. Front leg; **f**. Middle leg; **g**. Hind leg; **h**. Labium; **i**. Labrum; **j**. Tarsal claw; **k**. Mandibles; **l**. Head; **m**. Maxilla; **n**. Antenna; **o**. Abdomen; **p**. Penis; **q**. Penis apex; **r**. Tegmen, lateral view; **s**. Tegmen, inner view; **t**. Meso- and metaventrite; **u**. Coxites. Scale bars: 1.0 mm (**a–d**); 0.5 mm (**e–g, l, o, t**); 0.1 mm (**h–k, m, n, p, r, s, u**).

*Afissa* was well defined in Tomaszewska and Szawaryn (2016). It can be distinguished from its related Asian genera by the following combination of characters: antenna longer than width of head (Fig. 1l); coxites much longer than wide (Figs 1u, 2u, 3u, 4u); mandibular incisor edge without teeth (Figs 1k, 2k, 3k, 4k); lateral margins of elytra most often not or hardly visible from above (Figs 1a, 2a, 3a, 4a) (sometimes visible from above but narrow); metanepisternum with inner margin simple, smooth (Figs 1b, 2b, 3b, 4b); and mid and hind coxae on hind margin smooth (Figs 1f, g, 2f, g, 3f, g, 4f, g).

#### Distribution.

South and South-east Asia.

### Afissa parabicrescens

Taxon classificationAnimaliaColeopteraCoccinellidae

Zhang, Zhu & Wang sp. nov.

1D2676A0-D039-58C9-B37B-510F0772C2E4

https://zoobank.org/42EC1EA8-1627-426E-839E-16134549B145

Figs 1, 6

#### Type material (46 exx.).

***Holotype*: China: Yunnan**: • ♂, Baoshan City, Tengchong City, Wuhe County, 24.8549°N, 98.7703°E, 2155 m, 18–19.VII.2025, sweeping net, Zhu Z.H. & Zhang X.N. leg. ***Paratypes*: China: Yunnan**: • 3♂♂, 3♀♀, Ximeng, Longtan, 900 m, 9–10.V.2008, Xingmin Wang et al. leg.; • 2♂♂, 2♀♀, Xishuangbanna, Jinuo Mt., 28.IV.2008, Xingmin Wang et al. leg.; • 1♂, Jingdong City, Ailao Mt., 1–3.X.2006, Xingmin Wang leg.; • 1♂, Puer City, Dadugang, 950 m, 26.IV.2008, Xingmin Wang et al. leg.; • 7♂♂, Zhenyuan, Anban, 4.X.2006, Xingmin Wang leg.; • 2♂♂, Yingjiang, Nabang, Tongbiguan, 1000 m, 22–23.V.2008, Xingmin Wang et al. leg.; • 1♂, Zhenkang, Mengdui, 18–19.V.2008, 1400 m, Xingmin Wang et al. leg.; • 11♂♂, 8♀, Puer City, Caiyanghe, 4.V.2008, Xingmin Wang et al. leg.; • 1♂, 1♀, Xishuangbanna, Menga, 12.V.2009, Shunxiang Ren et al. leg.; • 1♂, Xishuangbanna, Mengla, 1160 m, 29.IV.2008, Xingmin Wang et al. leg.; • 1♂, Xishuangbanna, Menghun, 4.V.2008, Xingmin Wang et al. leg.

#### Diagnosis.

This new species closely resembles *Afissa bicrescens* Dieke, 1947, but can be easily distinguished from it by elytral pattern, elytral punctation and tegmen. In *A. parabicrescens* sp. nov., elytron is mostly reddish, covered with black maculae arranged in 2–2–1 pattern; head reddish without macula; elytron with longitudinal row of large punctures along elytral suture (Fig. 1a); penis guide stout in lateral view, slightly longer than parameres (Figs 1r, 6). In *A. bicrescens*, elytron is mostly blackish with elytral maculae variable (Pang et al. 2012: fig. 3a–f); elytron without longitudinal row of large punctures along elytral suture (Fig. 3a); head with transverse black macula in middle (Fig. 3d); penis guide comparatively slender in lateral view, parameres about 2/3 length of penis guide (Figs 3r, 6).

**Figure 5.**
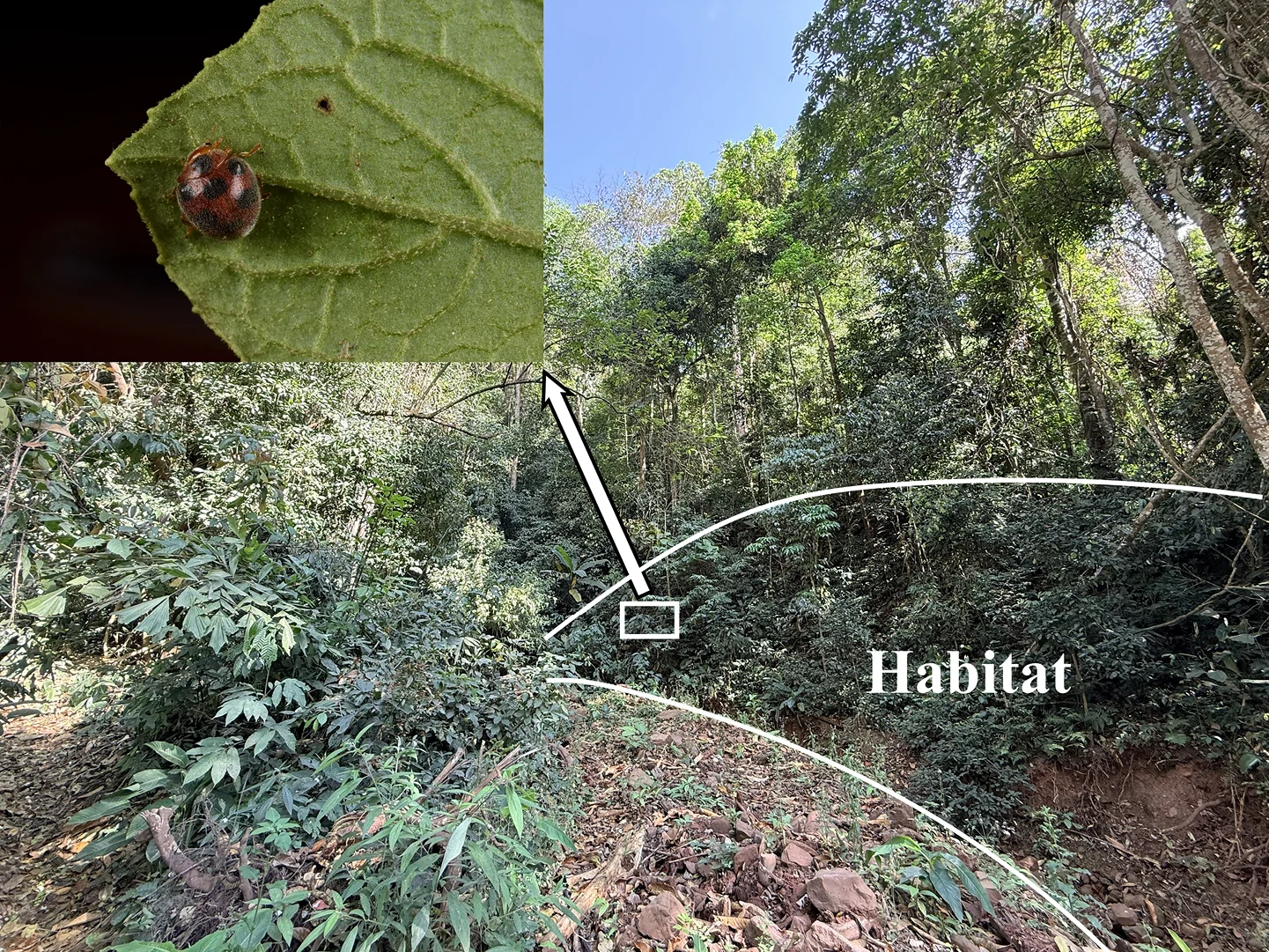
Yunnan Province, Xishuangbanna, habitat of Afissa cf. gedeensis.

**Figure 6.**
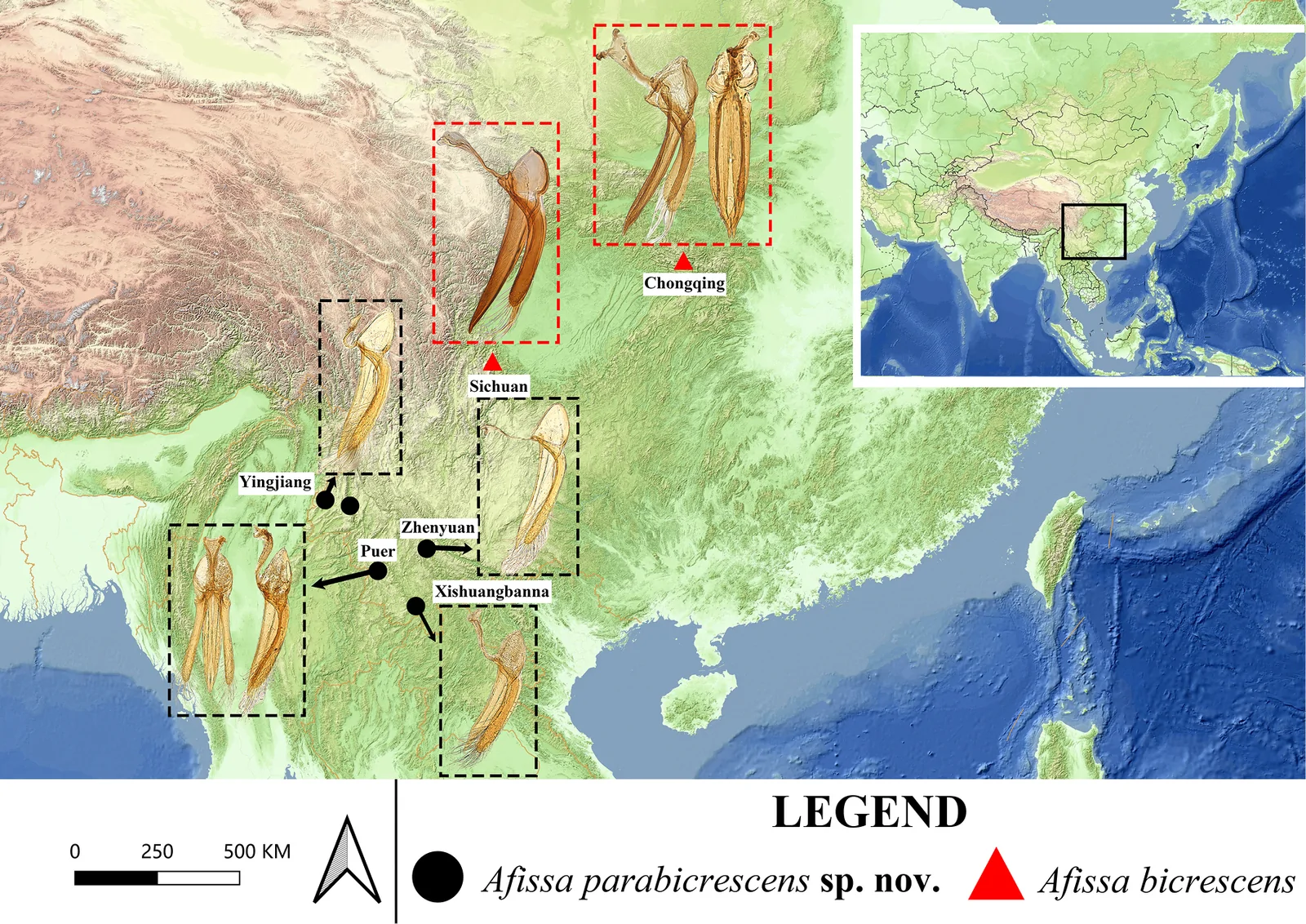
Distribution of *Afissa parabicrescens* sp. nov. and *Afissa bicrescens*.

#### Description.

***Measurements***. TL: 4.0–4.7 mm, TW: 3.0–3.6 mm, TH: 1.6–1.9 mm, TL/TW: 1.3, PL/PW: 2.1–2.4, EL/TW: 1.1.

Body outline oval in dorsal view (Fig. 1a), body medium-sized, moderately convex. Dorsum covered with dense, long and white setae. Elytron mostly reddish with black maculae arranged in 2–2–1 pattern, scutellar shield reddish. Pronotal disk with large, transverse and black macula; pronotal anterior and lateral margins yellowish-white, posterior margin reddish. Head yellowish. Ventral surface mostly yellowish except for meso- and metaventrite blackish (Fig. 1b).

Head ventrally flattened, with punctures dense and distinct (Fig. 1d). Eyes small and oval, coarsely faceted, eye canthus very small. Frons very wide. Clypeus emarginate in middle (Fig. 1l). Antenna composed of 11 antennomeres with 3-antennomere club, terminal antennomere with truncate apex, third antennomere elongate with subparallel margins (Fig. 1n). Labrum transverse, bearing sparse, small punctures and dense setae, posterior margin slightly emarginate (Fig. 1i). Mandible with two apical teeth, incisor edge smooth with dense setae, molar part without basal tooth (Fig. 1k). Maxilla with terminal palpomere securiform, lateral margins slightly explanate (Fig. 1m). Labium with terminal palpomere elongate and conical, nearly equal in length and width to penultimate one (Fig. 1h).

Pronotum widest at base, with punctures smaller than those on head. Pronotal lateral margins narrow and transparent, slightly upturned; hypomeron finely punctate. Prosternal process rectangular, apex slightly arcuate, with carinae inconspicuous and incomplete (Fig. 1b).

Elytron slightly explanate, elytral margins very narrow. Elytron with punctures varying in size, punctures mostly fine except for row of large punctures along elytral suture. Scutellar shield elongate and triangular (Fig. 1a). Epipleuron narrow with dense, long and white setae, ventrally flattened (Fig. 1b). Mesoventrite densely and moderately punctate, mesoventral anterior margin slightly emarginate. Metanepisternum with inner edge smooth. Anterior margin of metaventral intercoxal process straight; metaventral postcoxal lines complete, discrimen long but incomplete, almost reaching anterior margin of metaventral intercoxal process (Fig. 1t).

Legs long and slender, tarsi 4-segmented. Tibial apical half margin with pair of carinae, tibiae with apical spurs (Fig. 1e, f). Tarsal claw with two denticles, without basal tooth, inner denticle more robust but slightly shorter than outer one (Fig. 1j).

Abdomen with six ventrites. Abdominal postcoxal lines arcuate and incomplete. Ventrite 1 densely punctate, 2.0–3.0 diameters apart, anterior margin of abdominal intercoxal process nearly straight (Fig. 1o).

Penis simple, slightly curved. Penis capsule absent (Fig. 1p). Penis apex digitiform, without membranous region (Fig. 1q). Tegminal strut short, triangular at apex (Fig. 1s). Penis guide gradually tapering to apex in inner view, apex acute and bifurcated with deep cut. Penis guide in lateral view stout, upper margin nearly straight while lower margin arcuate (Figs 1r, 6. Parameres stick-shaped in lateral view, slightly curved, slightly shorter than penis guide, distal part of parameres with dense and very long setae.

Female genitalia (Fig. 1u) with spermatheca and infundibulum absent, coxites elongate and triangular, much longer than wide, distally setose with styli absent.

#### Distribution.

China (Yunnan) (Fig. 6).

#### Etymology.

The specific epithet *parabicrescens* refers to the similarity between this species and *A. bicrescens* in genital structure.

### Afissa longa

Taxon classificationAnimaliaColeopteraCoccinellidae

Zhang, Zhu & Wang sp. nov.

1BC01651-A9D7-53BD-AE91-A20F0DE17E23

https://zoobank.org/776427C7-F281-4EBC-AD3D-F239360E9616

Figs 2, 7

#### Type material (7 exx.).

***Holotype*: China: Yunnan**: • ♂, Baoshan City, Gaoligong Mt., Baihualing National Nature Reserve, 25.2938°N, 98.8083°E, 1400 m, 14.VII.2025, sweeping net, Zihao Zhu leg. ***Paratypes*: China: Yunnan**: • 1♂, Pingbian City, Lianhuatan, 900 m, 22–24.IV.2008, Xingmin Wang leg.; • 1♂, Ruili City, Nanjingli Village, 25.IX.2006, Xingmin Wang leg.; • 1♂, 1♀, Jinhong City, Jinuo Mt., 6.V.2009, Xingmin Wang et al. leg.; • 1♂, 1♀, Zhenkang City, Mengdui, 1400 m, 6.V.2008, Xingmin Wang et al. leg.

#### Diagnosis.

**Figure 7.**
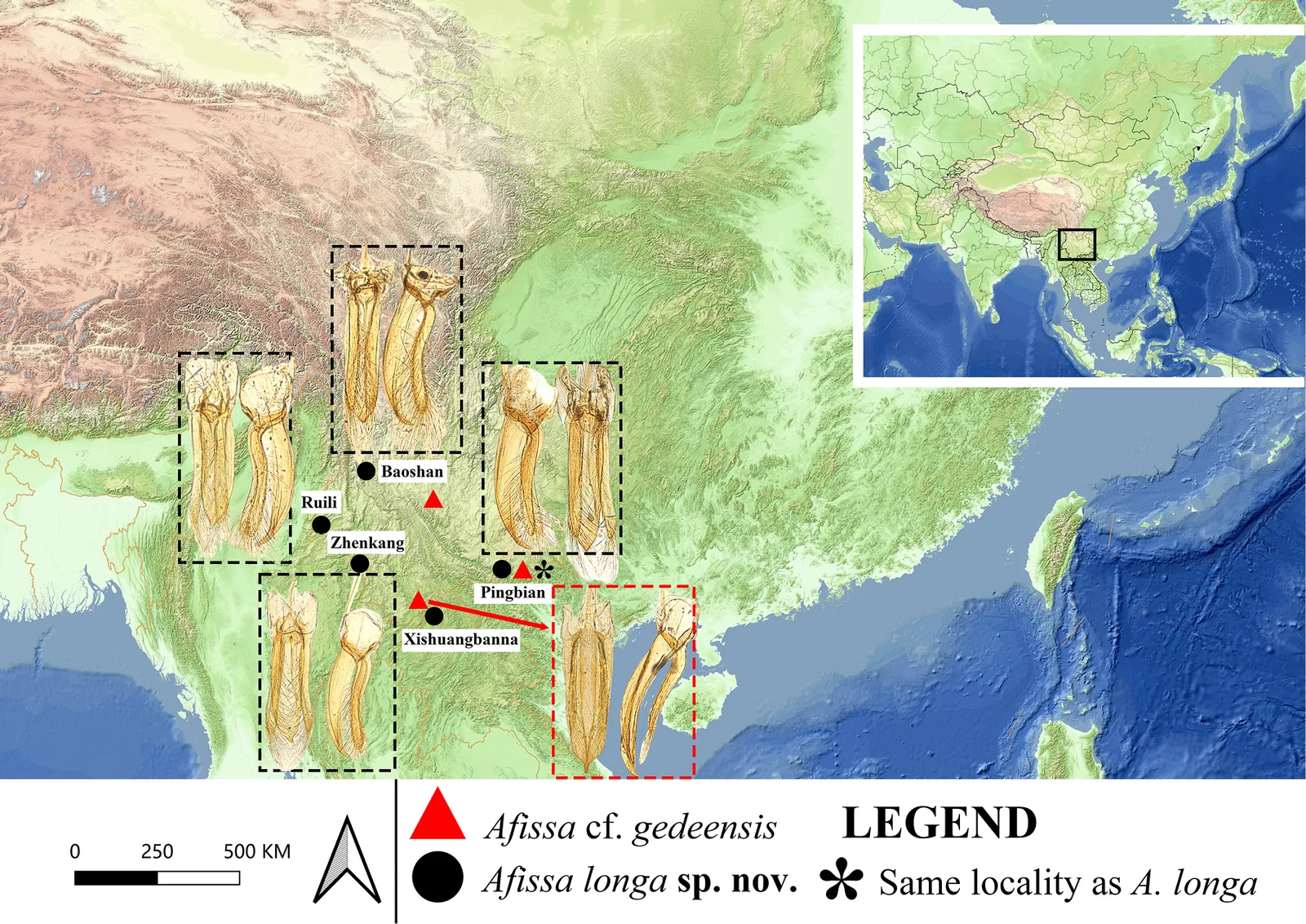
Distribution of *Afissa longa* sp. nov. and Afissa cf. gedeensis.

This new species is similar to *Afissa gedeensis* in elytral pattern but can be distinguished from it by the male genitalia. In *Afissa longa* sp. nov., penis capsule has outer arm distinctly elongate and enlarged (Fig. 2p); penis guide is robust and crescent-shaped in lateral view (Figs 2r, 7); and apex of penis guide is acute but comparatively short in inner view (Figs 2s, 7). In *A. gedeensis*, penis capsule has outer arm distinctly shorter; penis guide is comparatively slender in lateral view; apex of penis guide is acute and elongate in inner view (Dieke 1947, fig. 161; Pang et al. 2012, fig. 8e, f, g).

#### Description.

***Measurements***. TL: 4–4.8 mm, TW: 3.1–4.0 mm, TH: 1.9–2.1 mm, TL/TW: 1.2–1.3, PL/PW: 1.9–2.0, EL/TW: 1.0–1.1.

Body outline oval in dorsal view (Fig. 2a), body medium-sized, moderately convex (Fig. 2c). Dorsum with dense, long and white setae. Elytron mostly reddish with black maculae arranged in 2–2–1–1 pattern. Scutellar shield reddish. Pronotum reddish, pronotal disk with oval black maculae in middle (Fig. 2d). Head yellowish. Ventral surface mostly yellowish except for metaventrite blackish (Fig. 2b).

Head ventrally flattened, with moderately densely punctures. Eyes small and oval, coarsely faceted, eye canthus very small. Frons very wide. Clypeus emarginate in middle (Fig. 2l). Antenna composed of 11 antennomeres with 3-antennomere club, terminal antennomere with truncate apex, third antennomere elongate and distinctly divergent from its base to apex (Fig. 2n). Labrum transverse, with sparse and medium-sized punctures, densely setose (Fig. 2i). Mandible with three apical teeth, incisor edge smooth with dense setae, molar part without basal tooth (Fig. 2k). Maxilla with terminal palpomere securiform (Fig. 2m). Labium with terminal palpomere elongate and conical, nearly equal in length and width to penultimate one (Fig. 2h).

Pronotum widest at base, with punctures smaller than those on head, pronotal anterior angles angulate. Pronotal lateral margins narrow and transparent, slightly upturned (Fig. 2d). Prosternal hypomeron bears fine punctures. Prosternal process rectangular, apex slightly arcuate, with carinae inconspicuous and incomplete (Fig. 2b).

Elytron slightly explanate, elytral margins very narrow (Fig. 2a). Elytron with punctures varying in size, punctures mostly fine except for row of large punctures distributed from elytral anterior margin to distal part of elytron along suture. Scutellar shield elongate triangular (Fig. 2a). Epipleuron flattened and narrow with dense, long and white setae (Fig. 2b). Meso- and metaventrite with dense and medium-sized punctures, mesoventral anterior margin slightly emarginate (Fig. 2t). Anterior margin of metaventral intercoxal process straight; metaventral postcoxal lines complete, metaventral median discrimen long and nearly complete, almost reaching anterior margin of metaventral intercoxal process (Fig. 2t).

Legs (Fig. 2e–g) long and slender, tarsi 4-segmented. Tibial upper apical margin with pair of carinae, tibiae with apical spurs. Tarsal claw with two denticles, inner denticle more robust but shorter than outer one.

Abdomen with six ventrites (Fig. 2o). Abdominal postcoxal lines arcuate, nearly complete. Ventrite 1 with medium-sized and dense punctures, anterior margin of abdominal process nearly straight.

Penis elongate and slender, moderately curved (Fig. 2p). Penis capsule (Fig. 2p) with outer arm distinctly elongate and enlarged, inner arm short and small. Penis apex digitiform (Fig. 2q). Tegminal strut long and slender (Fig. 2r, s). Penis guide gradually tapering to apex in inner view, apex acute and slightly projected (Fig. 2s). Penis guide robust and crescent-shaped in lateral view, with outer margin arcuate while inner margin nearly straight, apex incurved and acute (Fig. 2r). Parameres slender and stick-shaped in lateral view, curved at base, shorter than penis guide (Fig. 2r).

Female genitalia (Fig. 2u) with spermatheca and infundibulum absent, coxites elongate and triangular, much longer than wide, distally setose with styli absent.

#### Distribution.

China (Yunnan) (Fig. 7).

#### Etymology.

The specific epithet *longa* refers to the elongate outer arm of the penis capsule; gender feminine.

#### Notes.

The shape of the penis guide in lateral view is consistent across different locations in this species (Fig. 7). However, specimens from Lincang and Ruili bear an asymmetric apex of the penis guide in inner view, whereas specimens from Pingbian and Baoshan are symmetric. The comparatively short apical projection of the penis guide in inner view is consistent among specimens.

### Afissa bicrescens

Taxon classificationAnimaliaColeopteraCoccinellidae

Dieke, 1947 stat. rev.

E4EA8684-4032-5A5C-904C-1CD993591C92

Figs 3, 6

Afissa bicrescens Dieke, 1947: 142, figs 96, 166, 210. Type locality: Szechwan (=Sichuan) Prov., China. Type depository: USNM (holotype).
Epilachna bicrescens : Pang and Mao 1979: 152, fig. 155; Ren et al. 2009: 266; Pang et al. 2012: 5, fig. 3; Wang and Chen 2022: 460, fig. 2.

#### Material examined (10 exx.).

**China: Chongqing**: • 2 ♂♂, Kaizhou District, Xuebaoshan Town, 1255 m, 29.VIII.2025, sweeping net, Wang Z.Z. leg.; **Sichuan**: • 4♂♂, 4♀♀, Baoxing, Fengyongzhai, 1500 m, 2–3.X.2007, Wang X.M. et al. leg.

#### Diagnosis.

*Afissa bicrescens* is morphologically similar to *Afissa uniformis* Pang & Mao, 1979 in body outline and elytral pattern, but can be easily distinguished from it by its male genitalia (Fig. 3p–s), especially the simple and slightly curved penis.

#### Redescription.

***Measurements***. TL: 3.3–4.6 mm, TW: 2.8–3.3 mm, TH: 1.5–1.8 mm, TL/TW: 1.2–1.4, PL/PW: 2.2, EL/TW: 1.1.

Body outline oval in dorsal view (Fig. 3a), body medium-sized, moderately convex (Fig. 3c). Dorsum with dense, long and white setae. Elytron mostly black with reddish variable maculae: (1) Longitudinal oval macula at anterior part of elytron, middle and distal part with reticulate macula (Fig. 3a); (2) Longitudinal narrow macula at anterior part, while transverse macula in middle, o-shaped macula occurs at distal part in addition (Pang et al. 2012: fig. 3d–f); (3) Elytron only with reticulate macula (Pang et al. 2012: fig. 3a–c). Pronotal disc black and margins brown (Fig. 3d). Head reddish with black transverse macula in middle, between eyes (Fig. 3d). Prosternum, epipleuron, meso- and metaventrite yellowish-brown fused with blackish maculae. Legs yellowish except for middle and hind femora with black maculae at basal part (Fig. 3b).

Terminal antennomere asymmetric with truncate apex (Fig. 3n). Mandible with at least three teeth visible, incisor edge smooth with dense setae; basal tooth absent (Fig. 3k). Maxilla with terminal palpomere securiform (Fig. 3m). Labium with terminal palpomere conical (Fig. 3h). Elytron slightly explanate, elytral margins very narrow (Fig. 3a). Elytron with dense punctures varying in size. Metaventral postcoxal lines complete, discrimen long and incomplete (Fig. 3t). Tibiae with apical spurs, tibial outer margins with longitudinal carinae (Fig. 3e–g). Tarsal claw with two denticles, inner denticle about half length of outer one, basal tooth absent (Fig. 3j). Abdominal postcoxal lines arcuate, nearly complete (Fig. 3o).

Penis simple, slightly curved (Fig. 3p). Penis capsule absent. Penis apex digitiform, without membranous region (Fig. 3q). Tegminal strut short, triangular at apex (Fig. 3r, s). Penis guide with outer and inner margin nearly parallel in lateral view, apex acute (Fig. 3r). Penis guide gradually tapering to apex in inner view, apex acute and bifurcated with deep cut (Fig. 3s). Parameres slender and stick-shaped in lateral view, about 2/3 length of penis guide, distal part with long and dense setae (Fig. 3r).

Female genitalia (Fig. 3u) with spermatheca and infundibulum absent, coxites elongate and triangular, much longer than wide, with terminal setae.

#### Distribution.

China (Shaanxi, Anhui, Sichuan, Chongqing, Hubei, Guizhou, Hunan) (Fig. 6).

#### Notes.

The illustrations of *A. bicrescens* provided in this research supported its generic position. *Afissa bicrescens* from Chongqing and Sichuan, as well as the line drawings in Dieke (1947), bear a similar penis guide which is elongate and slender in lateral view, while *A. bicrescens* figured by Pang and Mao (1979) as well as Pang et al. (2012) shows a comparatively robust penis guide in lateral view. Despite the differences in the penis guide, the parameres, which are distinctly shorter than the penis guide, are a consistent character in this species.

### Afissa cf. gedeensis

Taxon classificationAnimaliaColeopteraCoccinellidae

Dieke, 1947

D3E5E39B-48AA-5472-95D9-1A4A4B7347F8

Figs 4, 5, 7

Afissa gedeensis Dieke, 1947: 129, figs 94, 94A, 161. Type locality: Gede Mt., Java. Type depository: USNM (holotype). Tomaszewska and Szawaryn 2016: 52.
Epilachna gedeensis : Pang and Mao 1979: 141, fig. 140; Ren et al. 2009: 276; Pang et al. 2012: 10, fig. 8; Wang and Chen 2022: 468, fig. 4.

#### Material examined (15 exx.).

**China: Yunnan**: • 1♂, 1♀, Xishuangbanna Botanical Garden, 22.VIII.2005, Xingmin Wang et al. leg.; 1♂, 1♀, Xishuangbanna, Ganlaba, 20.IV.2002, Zhengqiang Peng leg.; • 1♂, Xishuangbanna, Mengla County, 23.VIII.2005, Xingmin Wang leg.; • 1♂, Dali City, Nanjian, 28.IX.2006, Xingmin Wang leg.; • 1♂, 1♀, Xishuangbanna, Jinuo Mt., 14.X.2006, Xingmin Wang leg.; • 2♂♂, 4♀♀, Xishuangbanna, Mengla County, 1160 m, 29.IV.2008, Shunxiang Ren et al. leg.; • 1♂, Pingbian City, Lianhuatan, 900 m, 22–24.IV.2008, Xingmin Wang leg.

#### Diagnosis.

This species highly resembles *Afissa hingstoni* Kapur, 1963, *Afissa bengalica* Dieke, 1947 and *Epilachna gorkhana* Miyatake, 1985 in male genitalia (Figs 4r, s, 7). Afissa cf. gedeensis can be distinguished from them by elytral pattern (Fig. 4a, c) and abdominal postcoxal lines (Fig. 4o). The elytron in A. cf. gedeensis bears black spots arranged in 2–2–1–1 pattern, the second row of spots may be joined into single transverse fascia; A. cf. gedeensis has abdominal postcoxal lines nearly complete. In *A. hingstoni* and *A. bengalica*, elytral pattern is made by the enlargement and connection of individual spots (Miyatake, 1985). In *E. gorkhana*, the abdominal postcoxal lines are incomplete and distinctly shorter than those in A. cf. gedeensis.

#### Description.

***Measurements***. TL: 4.8–5.6 mm, TW: 3.2–4.1 mm, TH: 1.8–2.0 mm, TL/TW: 1.4–1.5, PL/PW: 1.9–2.3, EL/TW: 1.1.

Body outline oval in dorsal view (Fig. 4a), body medium-sized, moderately convex (Fig. 4c). Dorsum with dense, long and white setae. Elytron reddish with black maculae arranged in 2–2–1–1 pattern, the second row of spots may be joined into single transverse fascia. Scutellar shield reddish. Pronotum reddish, pronotal disk with oval black maculae in middle (Fig. 4d). Head yellowish (Fig. 4d). Ventral surface mostly yellowish except for metaventrite blackish (Fig. 4b).

Mandible with apical teeth lamellar, incisor edge without teeth (Fig. 4k). Tarsal claw without basal tooth, outer denticle slender and distally erect; inner denticle robust, blade-shaped (Fig. 4j). Labium with terminal palpomere conical, nearly as long as penultimate one (Fig. 4h). Maxilla with terminal palpomere securiform, apex truncate (Fig. 4m). Abdomen with abdominal postcoxal lines nearly complete (Fig. 4o).

Penis elongate and slender, distal part recurved (Fig. 4p). Outer and inner arm weakly developed (Fig. 4p). Penis apex digitiform (Fig. 4q). Tegminal strut and tegmen elongate and slender (Fig. 4r, s). Penis guide moderately curved in lateral view, distally acute (Fig. 4r). Apex of penis guide bears elongate and acute protrusion in inner view (Fig. 4s). Parameres slender, distally setose, shorter than penis guide (Fig. 4r, s). Coxites (Fig. 4u) elongate and triangular, styli absent.

#### Distribution.

China (Yunnan) (Fig. 7).

#### Notes.

In the original descriptions and illustrations (Dieke 1947: fig. 94), *A. gedeensis* bears a reddish pronotum without any maculae, which is consistent with the descriptions in Pang and Mao (1979), who recorded *A. gedeensis* from China for the first time. However, the specimens of *A. gedeensis* illustrated by Pang et al. (2012) bear a large, transverse, black macula on the pronotal disc. Specimens examined in this study consistently bear a black macula on the pronotal disc but are significantly smaller than those in Pang et al. (2012). *Afissa gedeensis* was originally described from Java Island, and the wide geographic separation raises questions about the presence of *A. gedeensis* in China. These problems indicate the need for a revision of this species complex in the future.

## Discussion

In the present study, we further expand knowledge of the diversity of *Afissa* by describing two new species. However, numerous questions within the genus remain unresolved. The genus still involves many nomenclatural issues requiring clarification. After describing a series of new species, Dieke (1947) transferred many taxa previously placed in *Epilachna* to *Afissa*. In subsequent studies, species of both genera have been transferred to *Uniparodentata* and *Diekeana* (Tomaszewska and Szawaryn 2016). At present, it can only be confirmed that no members of *Epilachna* occur in China. With the examination of additional specimens of Epilachnini in future studies, numerous new combinations are expected to be proposed.

Many species of Epilachnini exhibit similar elytral patterns, and considerable intraspecific variation in elytral markings is also common within the tribe. Consequently, the validity of some species described solely from female specimens remains questionable. These issues will need to be addressed through lectotype designations, re-examination of type material, and the incorporation of molecular data. In recent years, DNA barcoding has increasingly been used in taxonomic studies of Coccinellidae for species identification (Poorani et al. 2023; Iqbal et al. 2024). Given its high diversity, Epilachnini would particularly benefit from application of this approach to species delimitation and for associating males and females.

Except for the male genitalia, the four species described and illustrated in this study share similar morphological characteristics of the legs, labium, labrum, maxilla, antenna, mesoventrite and metaventrite. However, they can be divided into two groups based on the morphology of the mandibles and tarsal claws. *Afissa parabicrescens* sp. nov. and *A. bicrescens* share elongate mandibular apical teeth and tarsal claws with the inner denticle shorter than the outer one (Figs 1J, 1K, 3J, 3K). In contrast, *Afissa longa* sp. nov. and A. cf. gedeensis possess well-developed, lamellar mandibular apical teeth and tarsal claws with the blade-shaped inner denticle more robust than the outer one (Figs 2J, 2K, 4J, 4K). These two groups differ distinctly in both mandibular and tarsal claw morphology. We speculate that variation in the physical properties of host plant leaves may influence feeding efficiency, thereby driving the evolution of the mandibles. Furthermore, differences in leaf surface characteristics may affect the ability of species to adhere to substrates, potentially promoting the diversification of tarsal claws. However, the feeding habits and host plant associations of many Epilachnini species remain poorly documented, limiting our understanding of these potential relationships. Further studies integrating ecological and morphological data are therefore needed to test these hypotheses.

## Supplementary Material

XML Treatment for Afissa

XML Treatment for Afissa parabicrescens

XML Treatment for Afissa longa

XML Treatment for Afissa bicrescens

XML Treatment for Afissa cf. gedeensis

## Additional information

### Artificial Intelligence (AI) use

The authors accept full responsibility for the content of the manuscript, including the disclosure of any use of AI.

No AI tools were used in the preparation of this manuscript.

### Author ORCIDs

X. Zhang https://orcid.org/0009-0004-3236-1158

Z. Zhu https://orcid.org/0009-0004-4656-831X

X. Wang https://orcid.org/0000-0001-9327-9159

### Data availability

All of the data that support the findings of this study are available in the main text.
